# BRD4 inhibition sensitizes renal cell carcinoma cells to the PI3K/mTOR dual inhibitor VS-5584

**DOI:** 10.18632/aging.103723

**Published:** 2020-10-13

**Authors:** Ming Xu, Lijun Xu, Yin Wang, Guangcheng Dai, Boxin Xue, Yuan-yuan Liu, Jianbing Zhu, Jin Zhu

**Affiliations:** 1 Department of Urology, the Second Affiliated Hospital of Soochow University, Suzhou, China; 2 Jiangsu Key Laboratory of Neuropsychiatric Diseases and Institute of Neuroscience, Soochow University, Suzhou, China; 3 Clinical Research and Laboratory Center, Affiliated Kunshan Hospital of Jiangsu University, Kunshan, China; 4 Department of Radiology, Suzhou Science and Technology Town Hospital, The Affiliated Suzhou Science and Technology Town Hospital of Nanjing Medical University, Suzhou, China

**Keywords:** renal cell carcinoma, PI3K/AKT/mTOR, VS-5584, BRD4, chemosensitization

## Abstract

Activation of the PI3K/AKT/mTOR pathway promotes the progression of renal cell carcinoma (RCC). This study tested the anti-RCC cell activity of the PI3K/mTOR dual inhibitor, VS-5584. We show that VS-5584 inhibited PI3K/AKT/mTORC1/2 activation in established (786-O and A498 lines) and primary RCC cells, thereby suppressing cell survival, proliferation, migration and cell cycle progression. VS-5584 induced significant apoptosis in RCC cells. A daily single oral dose of VS-5584 (20 mg/kg) significantly inhibited 786-O tumor growth *in vivo*. VS-5584 treatment of 786-O tumor xenografts and RCC cells resulted in feedback upregulation of bromodomain-containing protein 4 (BRD4). Furthermore, BRD4 inhibition (by JQ1 and CPI203), knockdown or complete knockout potentiated VS-5584-induced RCC cell death and apoptosis. Conversely, forced overexpression of BRD4 attenuated the cytotoxicity of VS-5584 in 786-O cells. Collectively, VS-5584 potently inhibits RCC cell proliferation and survival. Its anti-tumor activity is further enhanced by the targeted inhibition of BRD4.

## INTRODUCTION

Renal cell carcinoma (RCC) is the most common renal malignancy globally, causing significant human mortalities each year [1, 2]. In clinical practices, nephroureterectomy of the early-stage RCCs is yet the only curable treatment procedure [1]. However, a large proportion of RCC patients are diagnosed at advanced stages. Over 25% of them have local invasion and systematic metastasis [1, 3]. These patients often have a poor prognosis [1, 3].

Novel molecularly-targeted agents are needed for better RCC treatment [4, 5]. In RCC, PTEN depletion, PI3KCA mutation, and receptor tyrosine kinases (RTKs) overactivation will result in sustained activation of phosphoinositide 3-kinase (PI3K)-AKT- mammalian target of rapamycin (mTOR) cascade [6–9]. This signaling is essential for cancer cell proliferation and migration, as well as angiogenesis and chemo-resistance [6, 9–11]. This cascade is now an established and critical therapeutic target of RCC. Temsirolimus and everolimus, two mTOR inhibitors, are approved by the FDA for the treatment of curtained advanced RCC [6, 9–11]. Our group has previously shown that WYE-687, a AKT-mTORC1/2 inhibitor, potently suppressed RCC cell growth [12]. Recently, we demonstrated that a novel Akt inhibitor SC66 inhibited RCC cell progression, but through AKT-dependent and AKT-independent mechanisms [13].

VS-5584 is a potent dual inhibitor of PI3K and mTOR [14]. It displays almost equivalent activity against PI3K and mTOR [14]. This dual inhibitor exhibits certain pharmacokinetic properties. It is well-tolerated in animal studies [14]. The current study tested the anti-RCC cell activity of VS-5584.

Bromodomain-containing protein 4 (BRD4), a member of the BET (bromodomain and extraterminal domain) family [15], binds acetylated-histones to participate in epigenetic processes [16–18]. It is required for chromatin structure formation in daughter cells in mitosis. BRD4 recruits positive transcription elongation factor b and phosphorylates RNA polymerase II. It is an essential step for transcription elongation and expression of several key oncogenes, including Bcl-2 and c-Myc [17, 19].

In cancer cells BRD4 overexpression promotes cell survival, proliferation, and resistance to apoptosis [20]. Recent studies have proposed a pivotal function of BRD4 in chemoresistance. The BRD4 inhibitor JQ1 sensitized highly chemo-resistant pleural mesothelioma cells to cisplatin [21], and pancreatic cancer cells to gemcitabine [22]. The results of this study demonstrated BRD4 is a key resistance factor of VS-5584 in RCC cells.

## RESULTS

### VS-5584 inhibits survival, proliferation, cell cycle progression and migration in RCC 786-O cells

**Figure 1:**
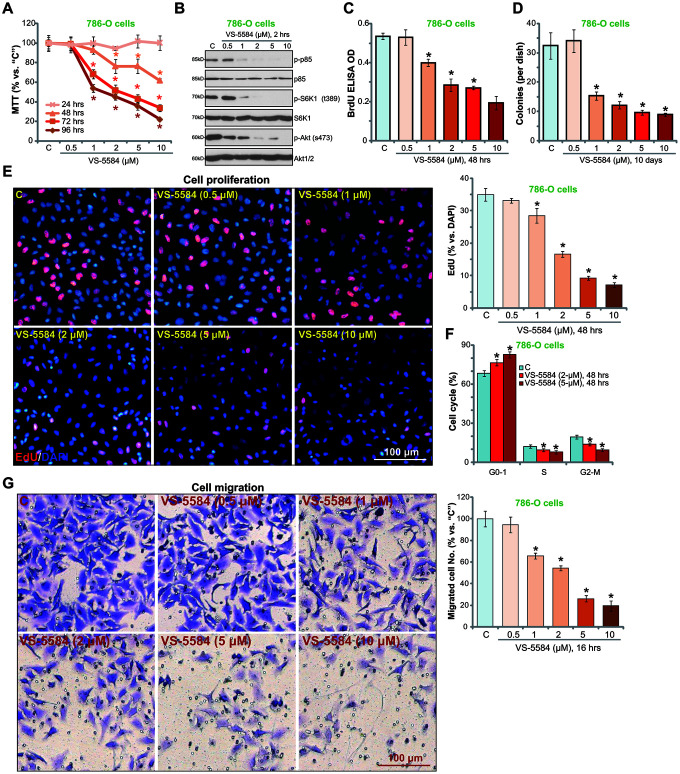
**VS-5584 inhibits survival, proliferation, cell cycle progression and migration in RCC 786-O cells.** RCC 786-O cells were either left untreated (“C”, same for all Figures), or treated with applied concentrations of VS-5584 (0.5-10 μM), cells were further cultured for the indicated time; Cell survival (**A**, MTT assay), PI3K-mTORC1/2 activation (**B**, Western blotting), cell proliferation (**C**–**E**, BrdU EILSA, soft agar colony formation and EdU incorporation staining assays) and cell cycle progression (**F**, PI-FACS) were tested, with cell migration examined by “Transwell” assays (**G**). For “EdU” assays, at least 800 cells in five random views were included to calculate EdU ratio for each treatment (same for all Figures). For “Transwell” assays five random views were included to calculate average number of migrated cells (same for all Figures). Data were presented as mean ± standard deviation (SD, n=5). *p< 0.05 *vs.* “C” group. The *in vitro* experiments were repeated four times, and similar results were obtained. Bar = 100 μm (**E**, **G**).

The current study tested the potential anti-tumor activity of VS-5584, a novel dual inhibitor of PI3K/mTOR [14, 23], in RCC cells. 786-O RCC cells [12, 24] were treated with different concentrations (0.5-10 μM) of VS-5584. MTT cell viability assay results showed that VS-5584 treatment inhibited 786-O cell survival in a dose- and time-dependent manner (Figure 1A). The IC_50_ of VS-5584 was between 1-5 μM (at 72/96-h treatment, Figure 1A). A lower dose of VS-5584 (0.5 μM) was unable to significantly inhibit 786-O cell viability (Figure 1A). Results in Figure 1B demonstrated that VS-5584 dose-dependently inhibited PI3K/mTORC1/2 cascade activation in 786-O cells. As shown in Figure 1B, treatment with VS-5584 inhibited the activation of phosphorylated (“p-”) p85, an indicator of PI3K activation, as well as of p-S6K1 (Thr-389) and p-Akt (Ser-473), which are substrates of mTORC1 and mTORC2 [25], respectively. The total protein levels of p85, S6K1, and Akt1/2 remained unchanged (Figure 1B).

To test cell proliferation *in vitro*, BrdU ELISA and soft agar colony formation assays were performed. VS-5584 treatment (1-10 μM) significantly decreased BrdU ELISA OD (Figure 1C) and the number of 786-O colonies (Figure 1D). These results indicated its anti-proliferative activity. Furthermore, VS-5584 dose-dependently inhibited EdU incorporation in 786-O cells (Figure 1E), further confirming proliferation inhibition.

Analysis of cell cycle distribution by PI-FACS showed that treatment with VS-5584 (2/5 μM) increased the percentage of cells in the G0/G1 phases, while decreasing the percentage of cells in the S and G2/M phases (Figure 1F). Testing cell migration *in vitro*, using “Transwell” assays, confirmed that VS-5584 (1-10 μM) reduced the number of migrated 786-O cells (Figure 1G). At the lowest concentration (0.5 μM), VS-5584 again failed to inhibit 786-O cell migration *in vitro* (Figure 1G). Treatment with vehicle control (dimethyl sulfoxide, 0.1-0.5%), as expected, had no significant effect on 786-O cell survival, proliferation and migration (Figure 1C–1G). These results show that VS-5584 inhibited survival, proliferation, cell cycle progression, and migration in RCC 786-O cells.

### VS-5584 induces apoptosis activation in RCC 786-O cells

**Figure 2:**
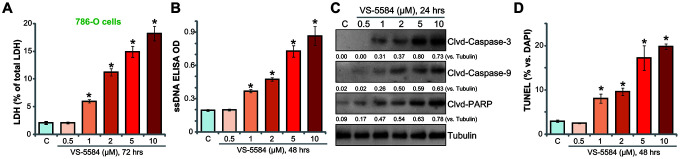
**VS-5584 induces apoptosis activation in RCC 786-O cells.** RCC 786-O cells were treated with applied concentrations of VS-5584 (0.5-10 μM), cells were further cultured for the indicated time; Cell death was tested by LDH medium release assay (**A**); Cell apoptosis was tested by ssDNA ELISA (**B**), Western blotting testing apoptosis proteins (**C**), and nuclei TUNEL staining (**D**). Data were presented as mean ± standard deviation (SD, n=5). *p< 0.05 *vs.* “**C**” group. The *in vitro* experiments were repeated four times, and similar results were obtained.

Cell death assay results showed that VS-5584 dose-dependently induced LDH release into the culture medium (Figure 2A), indicating cell death. VS-5584 treatment (1-10 μM) of 786-O cells also increased single strand DNA (ssDNA) production (Figure 2B). Western blotting assay results, Figure 2C, demonstrated that VS-5584 dose-dependently induced cleavages of caspase-3, caspase-9 and PARP (poly ADP-ribose polymerase) in 786-O cells. Additional studies demonstrated that the percentage of TUNEL-positive nuclei was significantly increased with VS-5584 (1-10 μM) treatment (Figure 2D), thereby confirming apoptosis activation. Lower concentrations of VS-5584 (0.5 μM) failed to induce 786-O cell apoptosis (Figure 2A–2D). Collectively, our data suggest that VS-5584 induced apoptosis activation in 786-O RCC cells.

### VS-5584 exerts anti-survival, anti-proliferative, and pro-apoptotic activity in the established and primary human RCC cells

**Figure 3:**
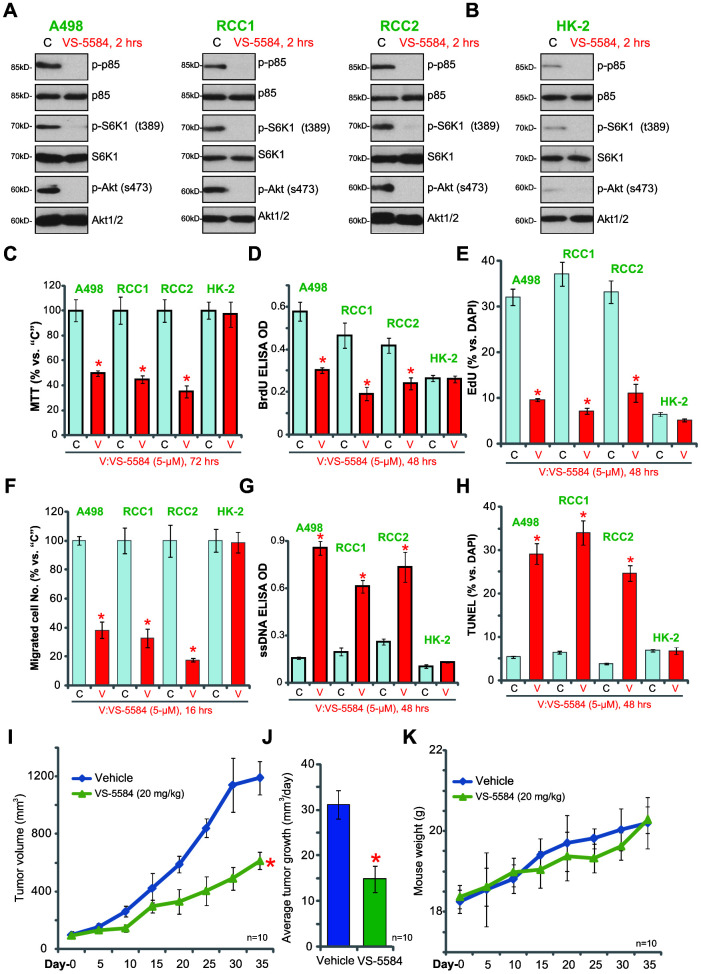
**VS-5584 exerts anti-survival, anti-proliferative, and pro-apoptotic activity in the established and primary human RCC cells.** A498 cells, the primary human RCC cells (“RCC1/RCC2”) or HK-2 renal epithelial cells were treated with VS-5584 (5 μM), cells were further cultured for indicated time; PI3K-mTORC1/2 activation (**A**, **B**, Western blotting), cell survival (**C**, MTT), proliferation (**D**, BrdU EILSA and **E**, nuclei EdU staining), migration (**F**, “Transwell” assay) and apoptosis (**G**, ssDNA ELISA and **H**, TUNEL staining) were tested. The 786-O xenograft tumor-bearing nude mice were administrated with vehicle control (“Vehicle”, saline), VS-5584 (20 mg/kg, oral administration, daily), the tumor volumes (**I**) and mice body weights (**J**) were recorded every five days for a total of 35 days; The estimated daily tumor growth was calculated (**K**); Data were presented as mean ± standard deviation (SD). *p< 0.05 *vs.* “**C**” group (**C**–**H**, n=5). *p< 0.05 *vs.* “Vehicle” (**I**, **J**, n=10). The *in vitro* experiments were repeated four times, and similar results were obtained. Bar = 100 μm (**E**, **F**, **H**).

The anti-tumor effects of VS-5584 were tested on the established human A498 RCC cells and two different primary human RCC cells, RCC1 and RCC2 (see our previous studies [13]). Western blotting results showed that activation of PI3K (“p-p85”), mTORC1 (“p-S6K1”), and mTORC2 (“p-Akt at Ser-473”) was inhibited by VS-5584 treatment (5 μM, 2 h) in A498 and primary human RCC cells (Figure 3A). The basal PI3K/mTORC1/2 activity was low in HK-2 renal epithelial cells (Figure 3B). Treatment with VS-5584 (5 μM) significantly inhibited the viability (MTT OD, Figure 3C) and proliferation (BrdU ELISA OD and nuclei EdU staining, Figure 3D, 3E) of A498 and primary RCC cells. Cell migration, tested by the “Transwell” assay, was largely inhibited in VS-5584-treated RCC cells (Figure 3F).

The ssDNA ELISA OD, an indicator of cell apoptosis, was increased in VS-5584-treated RCC cells (Figure 3G). To further confirm apoptosis activation we show that the ratio of TUNEL-positive nuclei was significantly increased with VS-5584 treatment in the RCC cells (Figure 3H). Whereas in HK-2 renal epithelial cells, the same VS-5584 treatment (5 μM) failed to inhibit cell survival (Figure 3C), proliferation (Figure 3D, 3E) and migration (Figure 3F). Nor did it induce apoptosis activation (Figure 3G, 3H). Thus, VS-5584 induced anti-survival, anti-proliferative, anti-migration and pro-apoptotic activities in established (A498) and primary human RCC cells.

To test the anti-RCC activity of VS-5584 *in vivo*, nude mice were subcutaneously inoculated with 786-O cells to form xenografts. Tumor growth curve analysis showed that a daily single dose of VS-5584 (20 mg/kg, oral administration) significantly inhibited 786-O tumor growth (Figure 3I). By calculating the estimated daily tumor growth, using the formula (tumor volume at day35− tumor volume at day0) ÷ 35, we show that 786-O xenograft growth *in vivo* was inhibited following treatment with VS-5584 (Figure 3J). The body weights of the experimental mice were not significantly different between the two groups (Figure 3K). There were no noticeable signs of apparent toxicity, suggesting that the VS-5584 treatment was well tolerated in the xenograft mouse model.

### BRD4 inhibition potentiates VS-5584-induced RCC cell death and apoptosis

**Figure 4:**
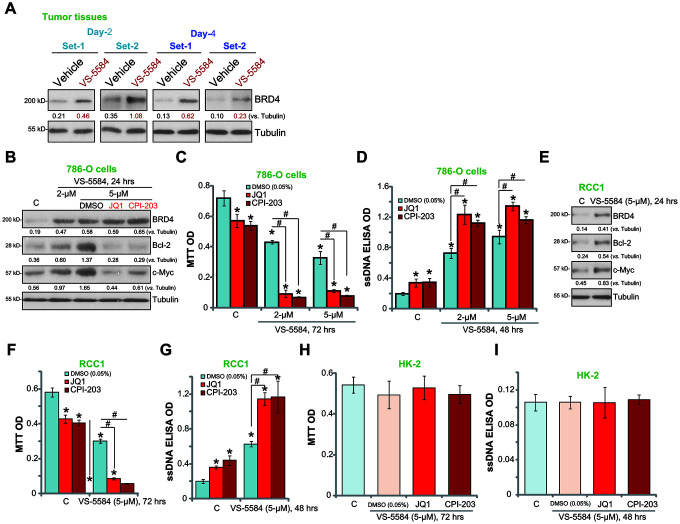
**BRD4 inhibition potentiates VS-5584-induced RCC cell death and apoptosis.** The 786-O xenograft tumor-bearing nude mice were administrated with vehicle control or VS-5584 (20 mg/kg, oral administration, daily), at treatment Day-2 and Day-4, 4 h after the VS-5584 or vehicle administration, two tumors (“Set-1/Set-2”) of each group were isolated, expression of BRD4 and Tubulin in tumor lysates was shown (**A**). 786-O cells (**B**) and primary human RCC cells (“RCC1”, **E**) were treated VS-5584 (or plus BRD4 inhibitors, **B**) for 24 h, listed proteins in total cell lysates were tested by Western blotting. 786-O cells (**C**, **D**), RCC1 primary cancer cells (**F**, **G**) or HK-2 cells (**H**, **I**) were pretreated with JQ1 (500 nM) or CPI203 (500 nM) for 30 min, followed by VS-5584 (2/5 μM) treatment for 48/72 h, cell survival and apoptosis were tested by MTT (**C**, **F**, **H**) and ssDNA ELISA (**D**, **G**, **I**), respectively. The listed proteins were quantified (**B**, **E**). Data were presented as mean ± standard deviation (SD, n=5). *p< 0.05 *vs.* “C” group. ^#^*p*< 0.05.

Although VS-5584 exerts anti-tumor effects against human RCC cells, its efficacy appears to be relatively low with an IC50 of 1-5 μM (Figures 1, 2), suggesting that RCC cells show resistance to VS-558. The BET family protein BRD4 is required for transcription elongation [17]. The BRD4-dependent proteins, Bcl-2 [26] and c-Myc [27, 28], are key oncogenic proteins. To examine the potential activity of BRD4 in chemoresistance, Western blotting was used to analyze BRD4 protein levels in tumor tissue lysates (Figure 3I). Results showed that BRD4 protein levels were significantly increased in VS-5584-treated 786-O tumor tissues compared with those in vehicle control-treated tumor tissues (Figure 4A). Therefore, VS-5584 administration *in vivo* induced BRD4 expression. Similarly, the protein levels of BRD4, Bcl-2, and c-Myc were increased in VS-5584 (2/5 μM)-treated 786-O cells *in vitro* (Figure 4B).

To confirm BRD4-induced RCC resistance to VS-5584, two known BRD4 inhibitors, JQ1 and CPI203, were utilized. Both BRD4 inhibitors blocked VS-5584 (5 μM)-induced Bcl-2 and c-Myc upregulation (Figure 4B). Furthermore, treatment with JQ1 and CPI203 significantly enhanced the ability of VS-5584 (2/5 μM) to decrease 786-O cell viability(Figure 4C) and to enhance apoptosis (Figure 4D). Treatment with JQ1 or CPI203 alone induced minor but significant 786-O cell death and apoptosis (Figure 4C, 4D).

In primary RCC cells (“RCC1”), VS-5584 treatment (5 μM, 24 h) induced feedback upregulation of BRD4, Bcl-2, and c-Myc (Figure 4E). Furthermore, treatment with JQ1 or CPI203 potently enhanced the cytotoxicity of VS-5584 in primary cancer cells (Figure 4F, 4G). Co-treatment with VS-5584 and the BRD4 inhibitors (JQ1/CPI203) failed to induce significant reduction in cell viability (Figure 4H) and apoptosis (Figure 4I) in HK-2 epithelial cells.

### BRD4 is the primary resistance factor of VS-5584 in RCC 786-O cells

**Figure 5:**
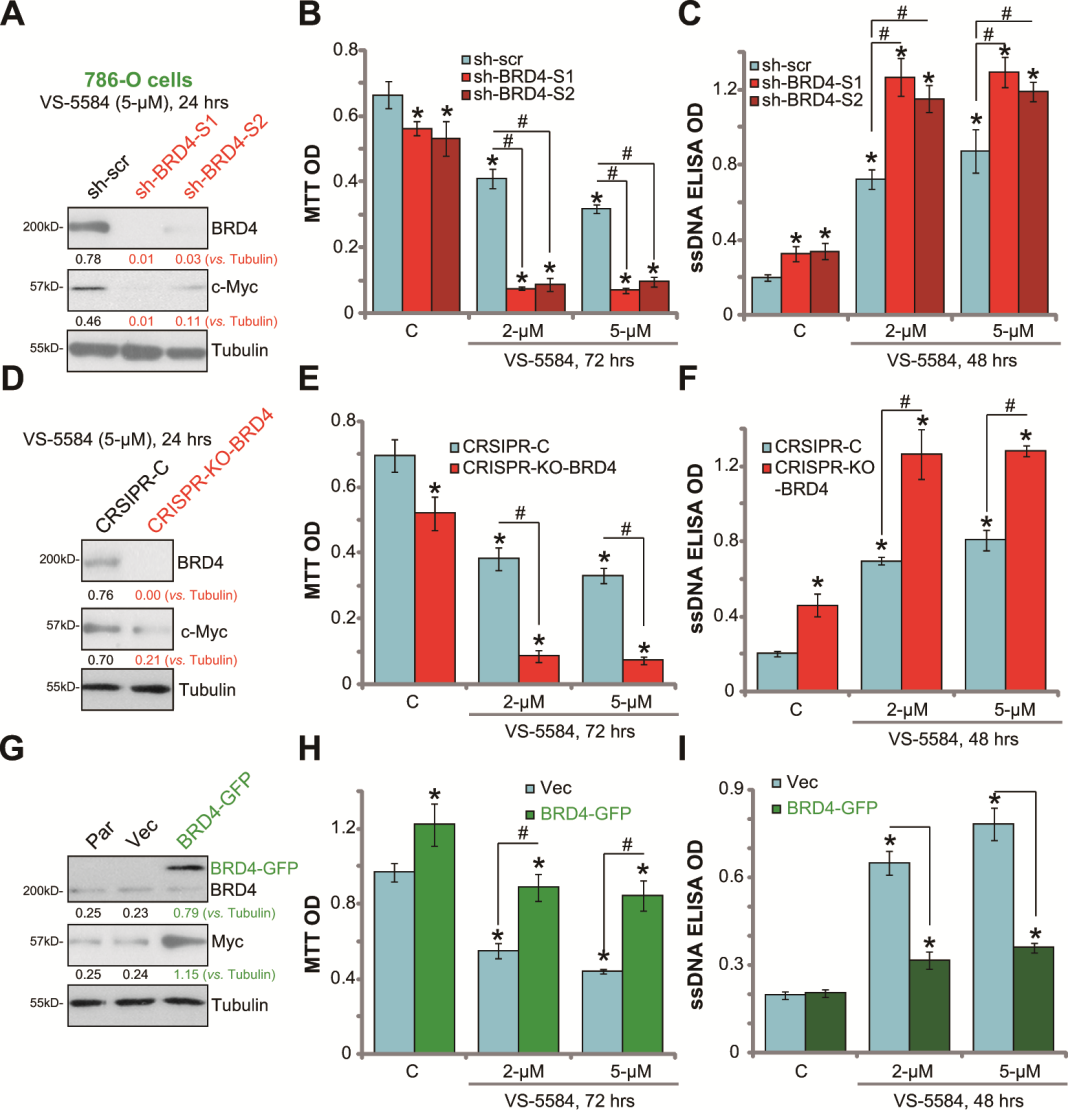
**BRD4 is the primary resistance factor of VS-5584 in RCC 786-O cells.** In VS-5584-treated stable 786-O cells with BRD4 shRNA (“sh-BRD4-S1/S2”, **A**–**C**), CRISPR-Cas9-BRD4-KO plasmid (**D**–**F**) or BRD4-expression vector (“BRD4-GFP”, **G**–**I**), BRD4, c-Myc and tubulin expression was shown (**A**, **D**, **G**). Cell survival and apoptosis were tested by MTT (after 72 h, **B**, **E**, **H**) and ssDNA ELISA (after 48 h, **C**, **F**, **I**), respectively. The listed proteins were quantified (**A**, **D**, **G**). Data were presented as mean ± standard deviation (SD, n=5). *p< 0.05 vs. “C” group. ^#^p< 0.05.

Because the pharmacological BRD4 inhibitors (JQ1 and CPI203) might have off-target toxicities, genetic strategies were employed to alter BRD4 expression in 786-O cells. Two lentiviral BRD4 shRNAs, with non-overlapping sequences (“sh-BRD4-S1/S2”), were transfected into 786-O cells. Western blotting results showed that the protein expression of BRD4, as well as the BRD4-regulated c-Myc gene were significantly downregulated by BRD4 shRNA treatment (Figure 5A). Importantly, 786-O cells transduced with BRD4 shRNA were more vulnerable to VS-5584 treatment, showing an increased viability reduction (Figure 5B) and apoptosis (Figure 5C).

To confirm BRD4 knockdown results, a CRISPR-Cas9-BRD4-KO plasmid was transfected into RCC 786-O cells to completely knockout BRD4 protein in the stable cells. In BRD4-KO cells, no BRD4 protein expression was observed even with VS-5584 treatment (5 μM, 24 h; Figure 4D). c-Myc expression was significantly decreased (Figure 5D). Compared with control cells, BRD4-KO 786-O cells were significantly more sensitive to VS-5584 (Figure 5E, 5F).

Based on the above results, we predicted that forced BRD4 overexpression shall inhibit VS-5584 activity. To test this hypothesis, a lentiviral BRD4-expression vector was transfected into 786-O cells. After puromycin selection, the stable cells showed exogenous BRD4 expression (tagged with GFP, Figure 5G). c-Myc expression was increased in BRD4-overexpressing cells (Figure 5G). Compared with the vector control cells, BRD4-overexpressing cells showed significant reduction in cell death (Figure 5H) and apoptosis activation (Figure 5I) following VS-5584 treatment. Collectively, these results confirm that BRD4 is the primary factor of VS-5584 resistance in RCC cells.

## DISCUSSION

There are two mTOR complexes, namely mTORC1 and mTORC2. mTORC1 inhibitors, such as everolimus, have been approved by the FDA for the clinical treatment of certain human RCCs [6, 9]. Yet, the clinical application of these inhibitors has several limitations. Rapamycin and its analogs can only partially inhibit mTORC1 activity [29, 30]. They fail to directly inhibit mTORC2, which is also important in the progression of RCC [7, 31].

VS-5584 is a novel PI3K/mTOR dual inhibitor, showing almost equivalent activity against PI3K and mTOR [14]. We found that VS-5584 blocked both mTORC1 and mTORC2 activation, as well as PI3K-Akt activity in RCC cells. We failed to observe feedback Erk-MAPK activation in VS-5584-treated RCC cells. A single daily oral dose of VS-5584 (20 mg/kg) significantly inhibited 786-O tumor growth *in vivo*. Hence, our data suggest that inhibition of the entirePI3K/AKT/mTOR cascade by VS-5584 could explain its superior anti-RCC cell activity.

Another important finding of this study was that BRD4, the BET family protein, is a key resistance factor against VS-5584 in RCC cells. VS-5584 treatment induced feedback upregulation of BRD4 in RCC cells, resulting in expression of BRD4 target proteins, Bcl-2 and c-Myc. Co-treatment with BRD4 inhibitors (JQ1/CPI203) potentiated VS-5584-induced RCC cell death and apoptosis. Furthermore, BRD4 knockdown or knockout enhanced VS-5584-induced cytotoxicity in RCC cells. Conversely, forced overexpression of BRD4 attenuated VS-5584-induced 786-O cell apoptosis.

The pharmacological and genetic evidence provided by this study indicate that BRD4 is a VS-5584 drug resistance factor in RCC cells. BRD4 inhibition may be an important strategy to sensitize RCC cells to VS-5584. The observed resistance to a PI3K-Akt inhibitor could be driven by the feedback activation of receptor tyrosine kinases (RTKs) [32]. It has been previously shown that BET inhibitors dissociated BRD4 from chromatin at the regulatory regions of multiple RTKs to reduce their expression level [32], thereby sensitizing a broad range of tumor cell lines to PI3K-Akt inhibitors [32]. Wang et al. demonstrated that BRD4 inhibition suppressed Sonic hedgehog signaling to sensitize pancreatic ductal adenocarcinoma cells to gemcitabine [22]. Moreover, JQ1 in combination with cisplatin induced synergistic inhibitory effects on human malignant pleural mesothelioma cells, possibly via the promotion of cell senescence and apoptosis [21]. Further studies are needed to explore the underlying mechanisms of BRD4 upregulation by VS-5584, and how BRD4 inhibition sensitizes RCC cells to VS-5584.

In summary, VS-5584 potently inhibits RCC cell proliferation and survival. Its anti-tumor activity is further enhanced by the targeted inhibition of BRD4.

## MATERIALS AND METHODS

### Chemicals and reagents

VS-5584, JQ1, and CPI203 were obtained from Sigma-Aldrich (St. Louis, MO). Cell culture reagents were purchased from Gibco (Grand Island, NY). The antibodies were purchased from Cell Signaling Technology (Danvers, MA). Puromycin was obtained from Sigma-Aldrich.

### Cell culture

Established human RCC cell lines (786-O and A498) as well as HK-2 human renal epithelial cells were obtained as described previously [13, 33]. The primary human RCC cells, derived from two different primary RCC patients (“RCC1” and “RCC2”, PTEN-null), were reported early [13]. The primary human cells were cultured in an appropriate medium as described previously [34].

### Methylthiazol tetrazolium (MTT) assay

Cells were seeded onto a 96-well tissue culture plate (3 × 10^3^cells per well). MTT assay was performed to test cell viability, according to the manufacturer’s instructions (Sigma-Aldrich). The MTT optical density (OD) at 590 nm was recorded.

### Soft agar colony formation assay

A total of 10,000 RCC 786-O cells per treatment were seeded on the top layer of 0.35% solidified agar in complete medium in 10-cm culture dishes, with the bottom layer containing 0.8% agar. VS-5584 was added to the complete medium and replaced every two days for a total of 10 days. Following this, colonies were stained with crystal violet (Sigma) and counted.

### BrdU (5-bromo-2-deoxyuridine) enzyme-linked immunosorbent assay (ELISA)

Cells were seeded onto 96-well tissue culture plates (3 × 10^3^ cells per well). The BrdU ELISA kit (Roche Diagnostics, Basel, Switzerland) was utilized to test cell proliferation *in vitro*. The BrdU ELISA absorbance at 405 nm was recorded.

### Cell cycle assay

The propidium iodide (PI; Invitrogen, Carlsbad, CA) flow cytometry assay was applied to test cell cycle distribution. Cells were seeded onto 6-well tissue culture plates (2 × 10^5^ cells per well). Following the applied treatment, cells were washed, fixed, and incubated with DNase-free RNase and PI. Cells were tested using a FACSCalibur instrument (BD Biosciences, Shanghai, China).

### *In vitro* cell migration assay

As described human RCC cells or the HK-1 cells (4 × 10^4^ cells of each condition in 200 μL serum-free medium) were seeded on the upper surfaces of “Transwell” chambers, coated with Matrigel (Sigma) [35, 36]. The lower compartments were filled with FBS-containing complete medium. Following incubation, the migrated cells to the lower chambers were fixed, stained and counted.

### EdU assay of cell proliferation

RCC cells or the HK-1 cells (1 × 10 ^5^cells/well) were seeded onto the six-well plates. An EdU (5-ethynyl-20-deoxyuridine) Apollo-488 *In Vitro* Imaging Kit (Ribo-Bio, Guangzhou, China) [37] was applied to examine and quantify cell proliferation. In brief, EdU (2.5 μM) dye was added to RCC cells or the HK-1 cells for 6-8h. Cell nuclei were co-stained with DAPI for 15 min, and visualized via a fluorescent microscope (Leica).

### Lactate dehydrogenase (LDH) assay for cell death

Cells were seeded onto 6-well tissue culture plates (2 × 10^5^cells per well). Cell death was examined by measuring the LDH content in the medium, using a 2-step enzymatic reaction LDH assay kit (Takara, Tokyo, Japan). Percentage of LDH release = LDH released in conditional medium ÷ (LDH released in conditional medium + LDH in cell lysates).

### Terminal deoxynucleotidyl transferase dUTP nick-end labeling (TUNEL) assay

As described previously [33], cells were seeded onto 6-well tissue culture plates (2 × 10^5^cells per well). TUNEL *In Situ* Cell Death Detection Kit (Roche Diagnostics, Shanghai, China) was utilized to quantify the number of TUNEL-labeled apoptotic nuclei.

### Western blotting

After the applied treatment, cells were treated with lysis buffer [38]. The total cell protein lysates (30 μg per treatment) were analyzed. Western blotting was performed following a previously described protocol [33]. Protein bands were visualized using enhanced chemiluminescence (ECL) reagents (Pierce, Suzhou, China), and quantified using the ImageJ software (National Institutes of Health).

### Single stranded DNA (ssDNA) ELISA

ssDNA accumulation is a characteristic marker of cell apoptosis. For each treatment, 30 μg of cell lysate (using the lysis buffer for western blotting) was analyzed. A ssDNA ELISA kit (Roche Diagnostics) was utilized to quantify DNA fragmentation. The ssDNA ELISA absorbance was recorded at 450 nm.

### BRD4 shRNA

Two different lentiviral BRD4 shRNAs, with unique and non-overlapping sequences (“S1/S2”), were provided by Dr. Zhao [39]. 786-O cells were seeded onto 6-well tissue culture plates (2 × 10^5^cells per well). Cells were transfected with BRD4 shRNA lentivirus for 24 h. Puromycin (2 μg/mL) was then used to select stable cells (4-5 passages). BRD4 knockdown in the stable cells was confirmed by Western blotting. Control cells were transfected with lentiviral scramble control shRNA (Santa Cruz Biotechnology).

### Exogenous BRD4 overexpression

The pSUPER-puro-BRD4-GFP expression vector was provided by Dr. Zhao [39], and was transfected into HEK-293T cells together with the viral packaging proteins VSVG and Hit-60 (Promega, Shanghai, China). After 48 h, the medium containing the virus particles was filtered, and 786-O cells were incubated in this medium for additional 48 h. Puromycin was used to select the stable cells (4-5 passages). Exogenous BRD4 overexpression in stable cells was confirmed by western blotting.

### BRD4 knockout (KO)

The CRISPR/Cas9 BRD4 KO plasmid (sc-400519-KO-2; Santa Cruz Biotechnology) was transfected to 786-O cells using Lipofectamine 2000 reagent (Invitrogen, Shanghai, China), and selected with puromycin after 4-5 passages. Control cells were treated with an empty vector with control small guide RNA (sgRNA; Santa Cruz Biotechnology). BRD4 expression in stable cells was tested by western blotting.

### Xenograft assay

The female nude mice were provided by the Animal Center of Chinese Academy of Science (Shanghai, China). 786-O cells were injected subcutaneously (*s.c.*) to the flanks of the nude mice. Within 20 days subcutaneous xenografts were established (around 100 mm^3^). Mice (n=10 each group) were treated with VS-5584. Mice body weight and bi-dimensional tumor measurements were taken every five days for a total of 35 days [40]. The animal protocol was approved by the Ethics Committee of Wenzhou Medical University.

### Statistical analysis

Quantitative results were presented as mean ± standard deviation (SD). Results were compared by one-way analysis of variance (ANOVA) followed by Tukey's test (SPSS version 21.0, Chicago, IL). Values of *p*< 0.05 were considered as statistically significant.
